# Clinical and biochemical profile of COVID-19 patients admitted in a tertiary care hospital in Visakhapatnam, India during post unlock 2.0 – a retrospective study

**DOI:** 10.25122/jml-2021-0341

**Published:** 2022-02

**Authors:** Venkat Narayana Goutham Valapala, Nikhila Dasari, Viswa Kalyan Kolli, Murty Mandapaka

**Affiliations:** 1. Department of General Medicine, Gitam Institute of Medical Sciences and Research, Gandhi Institute of Technology and Management (Deemed to be University), Visakhapatnam, India; 2. Department of Respiratory Medicine, Gitam Institute of Medical Sciences and Research, Gandhi Institute of Technology and Management (Deemed to be University), Visakhapatnam, India; 3. Department of Biochemistry, Gitam Institute of Medical Sciences and Research, Gandhi Institute of Technology and Management (Deemed to be University), Visakhapatnam, India

**Keywords:** COVID-19, SARS-CoV-2, RT-PCR, Unlock 2.0, Covid 19 – coronavirus disease 2019, gm – gram, ng – nanogram, mg – milligram, l – liter, dl – deciliter, cumm – cubic millimeter, RT PCR – reverse transcription-polymerase chain reaction, WHO – World Health Organization, MOHFW – Ministry of Health and Family Welfare, India

## Abstract

There have been more than 31378143 confirmed coronavirus disease 2019 (COVID-19) cases in India. It was declared a pandemic by the World Health Organization (WHO) on March 11, 2020. Because the risk of severe COVID-19 is not consistent across all individuals, uncertainty is linked to disease development. COVID-19 results have been related to systemic inflammation as a predictor. In COVID-19, increased levels of inflammatory markers have been associated with cytokine storm, coagulopathy, and endothelial dysfunction. A significant amount of research suggests that these results have a role in the cause of death in individuals suffering from a severe form of COVID-19. We aim to show our experience of COVID-19 at GITAM Institute of Medical Sciences and Research (GIMSR), Visakhapatnam. We analyzed data on 558 patients admitted to our dedicated COVID hospital during post unlock (UL) 2.0 in India from August 2 to August 31, 2020. The mean age was 43.65 years; 69% of them were male. Using MoHFW India severity guidelines, 68.10% were mild, 18.64% were moderate, and 13.26% were severe cases. Fatigue (66.13%) was the most common complaint, followed by anosmia (63.80%), fever (57.53%), diarrhea (56.09%), shortness of breath (22.40%), and others. The most common preexisting comorbidity seen in our patients was diabetes mellitus and hypertension, respectively. Laboratory parameters revealed mean hemoglobin of 13.04±1.91 gm/dl, mean total leukocyte count of 7378.49±3229 cells/cumm, mean platelet count of 2.3±0.8 lakhs/cumm, mean erythrocyte sediment rate of 40±30 mm/hr, mean ferritin level of 335.96 ng/ml, mean D-dimer level of 794.88 ng/ml and mean CRP of 23.27 mg/l. Severity was associated with higher age, symptomatic presentation, elevated leucocytes, and elevated inflammatory markers.

## Introduction

India went through many lockdowns and unlocked phases during the pandemic [1]. Infectious diseases have plagued humanity and have been potential killers since prehistoric times. The COVID-19 pandemic has taken us unprecedentedly. Since it is a new disease, it has become a challenge for doctors. There is a lack of vast clinical data concerning SARS-COV-2 infection and, most importantly, the disease progression [2]. COVID-19, caused by SARS-COV-2, began as an outbreak of respiratory tract infection of unknown cause and subsequently spread throughout the world [3].

It was declared a global pandemic on March 11, 2020, by the World Health Organization (WHO) [4]. The impact of COVID-19 on the population worldwide has created enormous social, environmental, health, and economic challenges. These countries are now trying to slow down the spread of the virus through social isolation, lockdowns, and increased testing and treatment facilities. For example, India has had four lockdowns (March 25, 2020–May 31, 2020) and two unlock periods (June 1–July 31, 2020).

Our understanding of COVID-19 has been evolving since its emergence. However, the most common presentation of this disease includes fever, fatigue, dry cough, muscle pain, breathlessness, and in some cases, headache, abdominal pain, diarrhea, nausea, vomiting etc. In severe cases, patients may present with severe dyspnea requiring ventilatory support [5, 6]. Diagnosis is achieved through conventional RT-PCR and high-resolution computed tomography (HRCT) chest.

## Material and Methods

The present study was conducted as a retrospective medical record-based study at Gitam Institute of Medical Sciences and Research, a dedicated COVID tertiary care hospital in Visakhapatnam, South India. 558 patients were admitted to this hospital from August 2 to August 31, 2020, during post unlock 2.0. The detailed information was collected from the medical records during this period. This study included all patients of different age groups. Inclusion criteria were a positive report of SARS-COV2 RT- PCR or HRCT (with chest CT severity score >8). All patients who had retroviral or autoimmune diseases were excluded from the study.

In this retrospective observational study, 558 patients were categorized as mild, moderate, and severe as per Ministry of Health and Family Welfare (MOHFW) guidelines [7]. Sociodemographic variables such as age, gender, clinical characteristics, and laboratory parameters of the patients were obtained from the records.

### Statistical Methods

All data obtained were statistically analyzed using Microsoft Excel and the Statistical Package for Social Sciences (SPSS) software version 21.0. Numbers and percentages were used to express categorical variables. The mean, standard deviation, and median were used to represent continuous variables.

## Results

A total of 558 cases were evaluated during post unlock 2.0, and the detailed phases are shown in Table 1 [1]. During post unlock 2.0, severe cases began to slowly decline in India.

**Table 1.** Lockdown and unlock phases in India due to COVID-19 [1].

**Phases**	**Start**	**End**
**Pre-lockdown (PL)**	1 January 20	24 March 20
**Lockdown 1.0 (LD1.0)**	25 March 20	14 April 20
**Lockdown 2.0 (LD2.0)**	15 April 20	3 May 20
**Lockdown 3.0 (LD3.0)**	4 May 20	17 May 20
**Lockdown 4.0 (LD4.0)**	18 May 20	31 May 20
**Unlock 1.0 (UL1.0)**	1 June 20	30 June 20
**Unlock 2.0 (UL2.0)**	1 July 20	31 July 20

**Figure 1.**
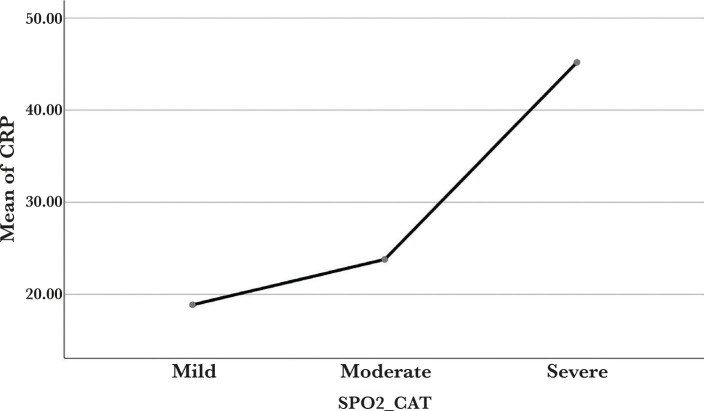
Mean plot of CRP (C-Reactive Protein) based on (Spo2) oxygen saturation.

380 (68.10%) cases were mild, 104 (18.64%) were moderate, and 74 (13.26%) were severe based on oxygen saturation during admission. This was based on MOHFW, India guidelines. The majority of the patients were male, 385 (69%), with the remainder being female, 173 (31%). The study population was separated into three groups, *i.e.*, mild (spo2>=95%), moderate (spo2 range between 90–94%), and severe(spo2<90%), as shown in Table 2. The mean age in our study was 43.65±16.211 years. The most common symptoms patients complained of were fatigue 369 (66.13%), followed by anosmia 356 (63.80%), fever 321 (57.53%), diarrhea 313 (56.09%), shortness of breath 125 (22.40%), and others. The most significant underlying comorbidity in our patients was diabetes mellitus in 162 patients and hypertension in 157 patients, respectively. Laboratory parameters revealed mean hemoglobin of 13.04±1.91 gm/dl, mean total leukocyte count of 7378.49 cells/cumm, mean platelet count of 2.3±0.8 lakhs/cumm, mean erythrocyte sediment rate of 40±30 mm/hr, mean ferritin level of 328.5, mean D-dimer level of 794.88 ng/ml and mean CRP of 23.27 mg/l as shown in Tables 3, 4 and Figure 1.

**Table 2.** Distribution of COVID-19 patients by sex and severity.

			**Mild**	**Moderate**	**Severe**
**Sex**	**Male**	**Count**	253	81	51
		**% within Sex**	65.7%	21.0%	13.2%
		**% within SPO2**	66.6%	77.9%	68.9%
	**Female**	**Count**	127	23	23
		**% within Sex**	73.4%	13.3%	13.3%
		**% within SPO2**	33.4%	22.1%	31.1%
**Total**		**Count**	380	104	74
		**% within Sex**	68.1%	18.6%	13.3%
		**% within SPO2**	100.0%	100.0%	100.0%

spo2 – oxygen saturation during admission.

**Table 3.** Mean and standard deviation of different variables.

	**N**	**Minimum**	**Maximum**	**Mean**	**Std. Deviation**
**Age (years)**	558	1	85	43.65	16.211
**SPO2 (%)**	558	80	99	94.85	4.523
**TLC (cells/cumm)**	558	1700	22400	7378.49	3229.382
**Platelets (Cells/cumm)**	558	.43	5.70	2.3559	.88049
**ESR (mm/hr)**	558	5	147	40.20	30.899

TLC – total leucocyte count; mm – millimeter; cumm – cubic millimeter; ESR – erythrocyte sedimentation rate; hr – hour.

**Table 4.** The interquartile range of ferritin and d-dimer in COVID-19 patients based on severity along with Kruskal-Wallis H test analysis.

	**Median (IQR)**			**Kruskal-Wallis H**	**P-Value**
	**Mild**	**Moderate**	**Severe**		
**Ferritin**	178 (112–293)	432 (216–653)	654 (413–884)	134.567	<0.001
**D-Dimer**	334 (202–479)	911 (387–1487)	1666 (928–2088)	152.627	<0.001

## Discussion

Out of 558 patients admitted to our COVID hospital in the given period, 380 (68.10%) were mild, 104 (18.64%) were moderate, and 74 (13.26%) were severe based on oxygen saturation during admission. In comparison, according to published data, roughly 80% of COVID-19 pneumonia symptoms were mild, 15% were moderate, and 5% were severe during the post unlock 2.0 phase in India [8, 9]. Most patients were admitted primarily after RTPCR, but many COVID-19 cases were admitted based on CT severity score, even if the RT PCR results were negative, because only 63% of affected cases have a positive RTPCR result, as per a study by Wenling Wang [10].

We found that 69% of the admitted cases among the affected individuals were males, whereas no deaths were reported in children. Children suffering from COVID-19 had better outcomes, which could be attributable to the absence of fully developed cellular and humoral immune responses [11]. It is hypothesized that single-cell RNA expression profiling of a cellular receptor of SARS-CoV 2, may play a significant role since Asians, especially in males, have a high number of angiotensin-converting enzyme 2 expressing cells in their lungs, making them susceptible to mortality [12].

Among the symptoms patients presented to the hospital, the most encountered was fatigue (66.13%) followed by anosmia (63%), fever (57%), cough (56.27%), diarrhea (56.09%). The prevalence of dyspnea was high in severe cases (39.2%). Fever was the most evident symptom of COVID-19 pneumonia, similar to the findings published in recent medical journals [13].

In comparison, higher values of CRP level were witnessed in severe cases than mild cases. Similarly, markers like serum ferritin levels and D-dimer levels are more elevated in most severe cases. In addition, platelet count was decreased in a few severe cases. According to our research, diabetes and hypertension were found in approximately 29.03% and 28.14% of the cases, respectively. COVID-19 patients are at risk of sequelae, including mortality, due to abnormal immune response, particularly T-cell response, heightened inflammatory response, hypercoagulable condition, and related comorbidities such as obesity, coronary artery disease (CAD), and chronic kidney disease (CKD). COVID-19 commonly affected people suffering from hypertension (HTN), diabetes mellitus (DM), and cardiovascular disease (CVD) and were associated with 13%, 5%, and 4% of COVID-19 patients in China’s Journal of Epidemiology [14]. According to a meta-analysis of more than seven trials that included 46,248 COVID-19 patients, HTN, DM, and cardiovascular diseases were all found to have a prevalence of 17%, 8%, and 5%, respectively [15].

## Conclusion

Covid-19 infection poses a significant risk to the population across the world. It has resulted in a heavy burden on the healthcare system. We describe the data analysis of 558 COVID-19 patients admitted in a tertiary hospital during post unlock 2.0 in South India. We observed that middle-aged men with preexisting comorbidities such as diabetes mellitus and hypertension were more likely to develop severe COVID-19. Inflammatory markers like CRP, D dimer, and serum ferritin levels can be used to predict disease severity and need to be interpreted accordingly with the patients’ underlying medical condition. People should be encouraged to undergo testing as soon as they become symptomatic, as the delay leads to a poor prognosis. Investigations such as CRP, D dimer, ferritin should be quickly done after hospitalization to assess the prognosis.

## Acknowledgments

### Conflict of interest

The authors declare no conflict of interest.

### Ethical approval

This study was approved by the Ethics Committee of GITAM Institute Of Medical Sciences and Research, Visakhapatnam, India (approval ID: GIMSR/Admn./Ethics/approval/IEC-12/2021).

### Personal Thanks

The authors sincerely acknowledge the continuous support of the President (GITAM SOCIETY), Secretary (GITAM SOCIETY), Pro VC (Medical Sciences), and also Principal, Vice Principals, Medical Superintendent, Deputy Medical Superintendents of GIMSR in the conduct of this study.

### Authorship

VNGV, ND, VKK, MM conceived the idea for this paper. VNGV contributed to the methodology, VNGV and VKK contributed to writing the original draft. MM contributed to editing the manuscript, ND contributed to data collection, and VKK contributed to data analysis. All authors provided an overview of analysis, supported manuscript writing, read and approved the the final draft of the manuscript.
